# Cost-Effectiveness of Proton Therapy Compared With Photon Therapy in Breast Cancer

**DOI:** 10.1001/jamanetworkopen.2025.54888

**Published:** 2026-01-22

**Authors:** Sara-Lise Busschaert, Eva Kimpe, Thierry Gevaert, Mark De Ridder, Koen Putman

**Affiliations:** 1 Research Centre on Digital Medicine, Department of Public Health, Vrije Universiteit Brussel, Brussels, Belgium; 2 Department of Radiation Oncology, Universitair Ziekenhuis Brussel, Vrije Universiteit Brussel, Brussels, Belgium

## Abstract

**Importance:**

Proton therapy reduces radiation exposure to the heart, lungs, and contralateral breast compared with conventional photon-based breast radiotherapy. However, its clinical benefit—particularly relative to photon deep inspiration breath-hold (DIBH)—remains uncertain, and its high cost raises questions about cost-effectiveness.

**Objective:**

To evaluate the cost-effectiveness of proton therapy compared with photon-based radiotherapy, including DIBH and free breathing (FB), among patients with breast cancer in Belgium.

**Design, Setting, and Participants:**

This economic evaluation using state-transition microsimulation cost-effectiveness analysis was conducted between December 2024 and February 2025 from the Belgian health care payer perspective. The model incorporated patient-specific probabilities of complications, including cardiovascular disease, secondary malignant tumors, and radiation pneumonitis, derived from normal tissue complication probability models and representative national data. Deterministic and probabilistic sensitivity analyses were conducted. Participants were a simulated cohort of Belgian women with breast cancer, reflecting national incidence and survival data, stratified by baseline cardiovascular risk, smoking status, and age.

**Exposures:**

Comparison of proton therapy vs photon therapy delivered via DIBH or FB. Scenarios were analyzed based on tumor laterality, regional nodal irradiation, and varying cost assumptions for proton therapy.

**Main Outcomes and Measures:**

The primary outcome was incremental cost-effectiveness ratio (ICER), expressed in euros per quality-adjusted life-year (QALY) gained. Cost-effectiveness defined by a willingness-to-pay threshold of €45 000 per QALY.

**Results:**

The simulated cohort included 1 million patients (310 000 aged 60 to 69 years [31.0%]). Proton therapy was not cost-effective in any scenario under base case assumptions. The greatest potential benefit was observed in patients with moderate to high baseline cardiovascular risk receiving left-sided irradiation with regional nodal irradiation, with an ICER of €292 438 (95% CI, €290 598-294 299) per QALY for proton therapy compared with photon free breathing. Under the most optimistic cost scenario (full treatment capacity and a 15% reduction in proton therapy costs), proton therapy was cost-effective compared with photon free breathing in approximately 36 000 of 1 000 000 simulated patients (3.6%), but it was not cost-effective compared with photon deep inspiration breath-hold in any subgroup, with ICERs exceeding €700 000 per QALY across all comparisons.

**Conclusions and Relevance:**

Findings from this economic evaluation of proton therapy for breast cancer suggest that proton therapy is unlikely to be cost-effective for most Belgian patients under current cost conditions, except for a small subset of high-risk patients, especially when DIBH is available.

## Key Points

**Question:**

Is proton therapy cost-effective compared with photon-based radiotherapy, including deep inspiration breath-hold (DIBH), among patients with breast cancer in Belgium?

**Findings:**

In this economic evaluation using a state-transition microsimulation analysis with 1 million patients, proton therapy was not cost-effective compared with photon therapy under base case assumptions. In the most favorable cost scenario, it was cost-effective for only 3.6% of patients when compared with free breathing, and was not cost effective for any patients when compared with DIBH.

**Meaning:**

These findings suggest that under current cost conditions, proton therapy appears unlikely to be cost-effective for most patients with breast cancer, particularly when DIBH is an option.

## Teaser

This economic evaluation compares the cost-effectiveness of proton vs photon therapy for breast cancer treatment across various clinical scenarios.

## Introduction

Proton therapy offers dosimetric advantages over photon-based radiotherapy, primarily due to the Bragg peak phenomenon, which enables precise dose delivery to tumors while minimizing exposure to surrounding organs at risk.^1^ This feature has led to growing interest in proton therapy for breast cancer,^2,3,4^ particularly for reducing radiation exposure to the heart, lungs, and contralateral breast.^5^ However, the clinical significance of these advantages remains uncertain^6^ and existing evidence raises concerns about increased skin reactions. Most crucially, the key question is whether the potential clinical benefits will justify the substantial financial investment required to establish and operate proton therapy facilities.^3^

Given the relatively low incidence of serious complications following breast irradiation, proton therapy is presumed to be cost-effective only for selected patients.^7^ The greatest potential benefit is thought to lie in shielding the heart from radiation, making patients with a high baseline cardiovascular disease (CVD) risk and those whose photon therapy would result in a high mean heart dose (MHD) the most likely candidates.^8^ Early analyses suggested that proton therapy could be cost-effective for patients at high risk of CVD.^9,10^ However, these studies were based on data from patients treated between 1970 and 1996, when photon-based treatments delivered higher MHDs.^11^ Since then, MHDs have significantly declined, lowering the associated cardiovascular risks.^12,13^ A more recent analysis by Vega et al^14^ indicated that proton therapy may be cost-effective for patients with at least 1 cardiovascular risk factor, provided that photon therapy would deliver a MHD of 5 Gy or higher. Based on this threshold, the authors^14^ suggested that proton therapy may be cost-effective for patients with high cardiovascular risk who are undergoing left-sided radiotherapy with regional nodal irradiation (RNI). Further supporting this, Li et al^15^ highlighted that both MHD and baseline cardiovascular risk are key factors, with cardiac risk being the most decisive for cost-effectiveness.

These analyses, however, did not consider additional outcomes potentially benefiting from proton therapy, such as the reduced risk of secondary malignancies. Austin et al^16^ sought to address this gap by evaluating the cost-effectiveness of proton therapy in 16 patients with left-sided breast cancer using a dosimetric analysis. Their findings indicated that cost-effectiveness was heavily influenced by secondary malignant tumor risk, with proton therapy deemed cost-effective in only 1 patient due to an increased lung dose. However, the analysis^16^ assumed a low, uniform baseline CVD risk of 1.5%, without exploring how higher risks might impact cost-effectiveness. Consequently, the question of patient selection and the cost-effectiveness of proton therapy remains unresolved. Moreover, the Austin et al study^16^ differed from earlier analyses by incorporating deep inspiration breath hold (DIBH), an increasingly adopted cardiac-sparing technique. Although MHDs achieved with photon DIBH are generally not as low as those achieved with proton therapy, the technique comes at a significantly lower cost.^17^ This raises the question whether proton therapy provides sufficient additional cardiovascular protection to justify its cost, particularly when photon DIBH is available as an alternative.

This study aims to advance the evidence base by analyzing the cost-effectiveness of proton therapy in Belgium—a country with high breast cancer incidence and strong survival outcomes^18^—making it an ideal setting for this investigation. Building on lessons learned from previous research, the analysis includes the main health outcomes hypothesized to benefit from proton therapy.^5^ For a comprehensive comparison, proton therapy is evaluated against photon therapy both under free breathing (FB) and with DIBH. Unlike earlier studies using Markov models, this study employs state-transition microsimulation to better capture heterogeneity and systematically assess how factors, such as baseline CVD risk, influence cost-effectiveness. The integration of normal tissue complication probability (NTCP) models further enables personalized risk predictions of radiation-induced complications, enhancing the accuracy of risk and benefit evaluations. Additionally, this study leverages data from detailed time-driven activity-based costing (TD-ABC) analyses to ensure a precise and realistic cost comparison.

## Methods

This economic evaluation follows the Consolidated Health Economic Evaluation Reporting Standards (CHEERS) reporting guideline; as a modeling study using simulated cohorts, no human participants were involved, and institutional review board approval was not required per Belgian law.^19^ A state-transition microsimulation model was developed to evaluate the cost-effectiveness of proton therapy vs photon therapy for women in Belgium aged 40 to 79 years with early-stage breast cancer. The simulated population reflected national incidence and survival data, with individualized risk predictions of radiation-induced complications derived using NTCP models and patient characteristics from Belgian health data (eTable 2 in Supplement 1). The model accounts for the main complications identified by the Particle Therapy Cooperative Group Breast Cancer Subcommittee that proton therapy could mitigate, including CVD, secondary lung and contralateral breast cancer, and radiation pneumonitis.^5^ To evaluate how radiotherapy parameters impact proton therapy’s benefits and how these effects vary based on baseline patient characteristics, multiple treatment strategies were analyzed, considering laterality, RNI, and technique (DIBH vs FB). The model was implemented in SIMUL8 Professional version 28.0, build 4249 (Simul8 Corporation), built in accordance with International Society for Pharmacoeconomics and Outcomes Research and the Society for Medical Decision Making recommendations.. Analyses were performed between December 2024 and February 2025.

### Model Structure

The model simulates patient pathways across several health states, including postradiotherapy health, CVD, lung cancer, contralateral breast cancer, and mortality states for each condition, in addition to background mortality. Transitions between states occur on an annual cycle, tracking each patient’s health status, costs, and outcomes. The model captures patient history, enabling individuals to experience multiple conditions simultaneously (eg, CVD and contralateral breast cancer) and recurring CVD events. In line with clinical knowledge, radiation pneumonitis (grade ≥2) is considered a short-term complication, typically resolving within 1 year, and can occur in either healthy or diseased states.^20^ Additionally, the model incorporates the long-term risks of secondary malignant tumors and CVD, which can persist up to 20 years following radiotherapy, as documented in current clinical evidence.^21,22^

### Comparators

The following treatment scenarios were assessed: (1) left-sided irradiation with RNI comparing proton vs photon therapy under FB, (2) right-sided irradiation with RNI comparing proton vs photon therapy under FB, (3) left-sided irradiation without RNI comparing proton vs photon therapy under FB, (4) right-sided irradiation without RNI comparing proton vs photon therapy under FB, (5) left-sided irradiation with RNI comparing proton vs photon therapy under DIBH, (6) right-sided irradiation with RNI comparing proton vs photon therapy under DIBH, (7) left-sided irradiation without RNI comparing proton vs photon therapy under FB, and (8) right-sided irradiation without RNI comparing proton vs photon therapy under FB.

Each modality was modeled based on typical dose distributions to critical organs (eTable 1 in Supplement 1). All comparators involve whole breast irradiation because it remains the guideline-recommended standard of care.^23^ In our setting, partial breast irradiation is not routinely used, and limited long-term data on proton partial breast irradiation preclude reliable modeling. A simulated population of 1 million patients was generated for each treatment strategy and scenario.

### Statistical Analysis

#### Transition Probabilities

Individual risks of complications were calculated based on representative Belgian health data and NTCP models, selected in alignment with clinical studies and standard practices. For CVD risk, the NTCP model was derived from the Darby et al study^22^ on the cardiotoxic effects of radiotherapy, in line with previous research.^24^ Baseline CVD risk was calculated using the Systematic Coronary Risk Evaluation (SCORE2) algorithms (eTable 3 in Supplement 1),^25,26,27^ while the risk of recurrent events was estimated using the European Action on Secondary and Primary Prevention by Intervention to Reduce Events Risk Model (eTable 7 in Supplement 1).^28^ Both models are tailored for European populations. Age-specific CVD mortality rates were sourced from the SCORE2 project (eTable 6 in Supplement 1). Baseline lung cancer risks were calculated using the Tammemägi et al^29^ equations, selected for their accuracy in predicting lung cancer risk across smoking histories and proven applicability in multiple populations (eTable 4 and eTable 5 in Supplement 1). Subsequently, postradiotherapy risks were estimated using an NTCP model derived from a comprehensive meta-analysis of breast irradiation complications.^21^ Contralateral breast cancer risks were calculated using the Schneider model, which considers age and incorporates dose-dependent cell-killing effects and fractionation.^30^ Mortality risks for secondary cancers were sourced from the Belgian Cancer Registry (eTable 8 in Supplement 1). For calculating radiation pneumonitis (grade ≥2) risk, the Lyman-Kutcher-Burman model based on mean lung dose was selected due to its superior accuracy in breast cancer patients, as shown in a comparative analysis of multiple NTCP models.^31^ Finally, mortality risks from other causes, considering differential survival based on nodal status, were calculated from population-based analyses (eTable 9 in Supplement 1). Detailed information on the NTCP models and risk calculations is provided in eAppendix 1 in Supplement 1.

#### Utilities

Baseline utility values were obtained from a representative Belgian survey (eTable 10 in Supplement 1), while utility decrements for complications were sourced from a systematic review on health-related quality of life in chronic diseases (eTable 11 in Supplement 1). A utility value of 0 was assigned across all mortality states.

#### Costs

Treatment costs for photon therapy were sourced from a Belgian TD-ABC analysis,^17^ while proton therapy costs were obtained from a Dutch TD-ABC analysis, where this treatment was recently introduced with partial reimbursement, justified by its potential to reduce CVD risk (eAppendix 2 and eTable 12 in Supplement 1).^32^ The base case cost reflects the initial start-up phase; however, to anticipate future efficiencies, various cost scenarios were explored. These scenarios assumed an increase in treatment capacity, leading to potential cost reductions. An additional scenario considered further cost reductions at maximum capacity, driven by factors such as technological advancements. Cost calculations for complications followed Belgian guidelines and were performed from a health care payer perspective (eTable 13 in Supplement 1). All cost data were converted to 2024 Belgian euros using the Campbell and Cochrane Economics Methods Group Evidence for Policy and Practice Information and Coordinating Centre Cost Converter to maintain uniformity and enable accurate comparisons.^33^

#### Outcomes

The primary outcomes include incremental cost-effectiveness ratios (ICERs), incremental effects measured in quality-adjusted life years (QALYs), and incremental costs. In line with Belgian guidelines, QALYs were discounted at a rate of 1.5%, costs were discounted at 3.0% and a willingness-to-pay threshold of €45 000 was employed to evaluate cost-effectiveness. Deterministic and probabilistic sensitivity analyses were performed to assess the robustness of the model’s results under different assumptions and to evaluate how variations in key parameters affect the outcomes (eAppendix 3 in Supplement 1).

## Results

### Base Case

The simulated cohort comprised 1 000 000 individuals, including 170 000 aged 40 to 49 years (17.0%), 260 000 aged 50 to 59 years (26.0%), 310 000 aged 60 to 69 years (31.0%), and 250 000 aged 70 to 79 years (25.0%) (Table 1). In the base case, proton therapy was not cost-effective in any scenario. Proton therapy was consistently less cost-effective when compared with photon therapy using DIBH rather than FB, with ICERs exceeding €1 million per QALY in all 4 clinical scenarios (range, €1 067 379-€1 538 684), compared with €475 768 to €962 524 per QALY when compared with photon FB (Table 2).

**Table 1.** Base Case Results: Patient Characteristics Outcomes^a^

Patient subgroup	Patients, No. (%)	ICER, mean (95% CI), €								
		Left-sided irradiation				Right-sided irradiation				
		Without RNI		With RNI:		Without RNI			With RNI	
		Proton vs photon FB	Proton vs photon DIBH	Proton vs photon FB	Proton vs photon DIBH	Proton vs photon FB	Proton vs photon DIBH	Proton vs photon FB		Proton vs photon DIBH
Total	1 000 000 (100)	590 706 (504 907-710 950)	1 182 977 (904 264-1 706 656)	475 768 (407 082-571 915)	1 067 379 (799 066-1 604 409)	1 392 610 (985 715-2 363 810)	1 538 684 (1 038 750-2 953 698)	962 524 (757 085-1 319 494)		1 385 459 (1 011 293-2 194 798)
Age group, y										
40-49	170 000 (17)	2 373 228 (1 992 866-2 932 391)	3 177 180 (2 534 383-4 255 895)	1 326 641 (1 128 974-1 607 460)	1 765 723 (1 611 817-1 951 617)	3 169 947 (2 159 704-5 954 920)	3 339 341 (2 384 261-5 570 802)	1 395 681 (1 356 455-1 437 292)		1 833 425 (1 291 364-3 157 674)
50-59	260 000 (26)	1 204 661 (1 196 156-1 211 591)	2 282 169 (2 262 566-2 302 104)	889 863 (846 760-937 576)	1 562 074 (1 414 137-1 744 475)	2 600 627 (2 411 235-2 822 048)	3 065 763 (2 943 913-3 198 076)	1 532 256 (1 394 869-1 699 691)		2 143 171 (2 096 683-2 191 745)
60-69 y	310 000 (31)	€548 419 (514 586-586 894)	918 058 (907 347-929 002)	420 797 (395 357-449 667)	918 720 (806 139-1 067 614)	973 798 (939 233-1 010 979)	1 062 047 (1 025 008-1 101 852)	771 601 (729 519-818 799)		1 118 205 (1 031 045-1 221 395)
70-79	250 000 (25)	299 532 (294 048-305 222)	747 569 (745 930-749 213)	267 668 (263 247-272 238)	760 678 (680 469-862 301)	1 039 562 (1 023 694-1 055 914)	1 151 927 (1 124 309-1 180 902)	742 271 (640 189-882 829)		1 117 785 (907 717-1 454 758)
Smoking status										
Never	620 000 (62)	654 923 (637 533-673 287)	1 490 219 (1 437 009-1 547 476)	582 909 (575 417-590 598)	1 596 401 (1 590 325-1 602 522)	3 343 798 (3 251 609-3 441 348)	3 295 463 (3 165 070-3 437 050)	1 468 701 (1 391 632-1 554 750)		2 176 465 (2 130 137-2 224 867)
Ex-smoker	250 000 (25)	571 473 (528 048-622 656)	1 023 025 (894 016-1 195 378)	491 131 (456 346-531 590)	1 174 454 (943 042-1 555 532)	1 214 967 (1 097 785-1 360 100)	1 403 417 (1 127 791-1 857 331)	1 056 799 (960 060-1 175 085)		1 391 626 (1 297 225-1 500 815)
Current smoker	130 000 (13)	421 636 (417 432-425 906)	703 939 (685 393-723 448)	244 437 (240 531-248 462)	388 447 (385 418-391 523)	396 968 (373 810-423 108)	458 393 (418 169-507 061)	341 622 (322 803-362 714)		507 948 (494 279-522 389)
Baseline CVD risk										
Low	640 000 (64)	960 105 (893 387-1 037 560)	1 833 643 (1 769 178-1 902 935)	723 657 (713 069-734 561)	1 447 377 (1 386 127-1 514 288)	2 473 657 (2 424 442-2 524 881)	2 703 052 (2 505 470-2 934 378)	1 253 299 (1 147 799-1 380 179)		1 695 636 (1 508 119-1 936 323)
Moderate or high	360 000 (36)	346 774 (340 062-353 751)	719 500 (693 376-747 651)	292 438 (290 598-294 299)	723 603 (712 880-734 648)	775 857 (775 606-776 109)	863 220 (833 174-895 442)	677 300 (659 213-696 404)		1 041 416 (1 015 626-1 068 534)

Abbreviations: CVD, cardiovascular disease; DIBH, deep inspiration breath-hold; FB, free breathing; ICER, incremental cost-effectiveness ratio; RNI, regional nodal irradiation.

^a^ The results reflect the average model outcomes based on simulations of 1 million patients for each combination of radiotherapy modality (proton or photon), technique (DIBH or FB), and clinical scenario (left-sided radiotherapy, left-sided radiotherapy with RNI, right-sided radiotherapy, and right-sided radiotherapy with RNI).

**Table 2.** Base Case Results^a^

Treatment comparison	ICER, € per QALY, mean (95% CI)	Incremental QALYs, mean (95% CI)	Incremental costs, mean (95% CI), €	CVD risk reduction, % (95% CI)	Lung cancer risk reduction, % (95% CI)	Contralateral breast cancer risk reduction, % (95% CI)	Radiation pneumonitis risk reduction, % (95% CI)
Left-sided no RNI proton vs photon FB	590 706 (504 907-710 950)	0.0836 (0.0697-0.0976)	49 410 (49 294-49 526)	2.65 (2.48-2.83)	0.51 (0.39-0.63)	0.35 (0.30-0.39)	0.22 (0.19-0.26)
Left-sided no RNI proton vs photon DIBH	1 182 977 (904 264-1 706 656)	0.0417 (0.0290-0.0544)	49 344 (49 233-49 454)	0.96 (0.83-1.10)	0.46 (0.37-0.56)	0.36 (0.31-0.40)	0.19 (0.16-0.22)
Left-sided RNI proton vs photon FB	475 768 (407 082-571 915)	0.1021 (0.0851-0.1191)	48 576 (48 488-48 664)	2.27 (2.12-2.41)	1.23 (1.09-1.36)	3.21 (3.15-3.26)	1.91 (1.83-1.99)
Left-sided RNI, proton vs photon DIBH	1 067 379 (799 066-1 604 409)	0.0456 (0.0304-0.0608)	48 697 (48 620-48 774)	0.35 (0.21-0.49)	0.89 (0.78-1.00)	3.23 (3.18-3.28)	1.23 (1.15-1.31)
Right-sided no RNI proton vs photon FB	1 392 610 (985 715-2 363 810)	0.0359 (0.0212-0.0506)	50 026 (49 909-50 143)	0.09 (−0.06 to 0.24)	1.03 (0.91-1.14)	0.36 (0.31-0.40)	0.23 (0.21-0.26)
Right-sided no RNI proton vs photon DIBH	1 538 684 (1 038 750-2 953 698)	0.0321 (0.0168-0.0475)	49 467 (49 353-49 581)	0.09 (−0.06 to 0.25)	0.88 (0.76-0.99)	0.36 (0.31-0.41)	0.19 (0.17-0.22)
Right-sided RNI proton vs photon FB	962 524 (757 085-1 319 494)	0.0511 (0.0374-0.0649)	49 228 (49 153-49 302)	0.30 (0.16-0.44)	1.12 (1.00-1.23)	3.22 (3.18-3.27)	1.93 (1.85-2.01)
Right-sided RNI proton vs photon DIBH	1 385 459 (1 011 293-2 194 798)	0.0352 (0.0223-0.0482)	48 809 (48 727-48 890)	0.19 (0.05-0.32)	0.67 (0.58-0.76)	3.23 (3.17-3.28)	1.23 (1.15-1.30)

Abbreviations: CVD, cardiovascular disease; DIBH, deep inspiration breath-hold; FB, free breathing; ICER, incremental cost-effectiveness ratio; QALYs, quality-adjusted life years; RNI, regional nodal irradiation.

^a^ The results represent average model outcomes derived from simulations of 1 million patients for each combination of radiotherapy modality (proton or photon), technique (DIBH or FB), and clinical scenario (left-sided radiotherapy, left-sided radiotherapy with RNI, right-sided radiotherapy, and right-sided radiotherapy with RNI). For each outcome, 95% CIs are reported in brackets to indicate the range of statistical uncertainty.

Incremental QALYs were substantially lower due to smaller reductions in cardiopulmonary complications. The relative change in ICER was greatest in the scenario where proton therapy provided the largest benefit over photon FB—left-sided irradiation with RNI (a 124.4% increase)—and smallest in the scenario where proton therapy benefits were least—right-sided irradiation without RNI (10.5% increase).

However, cost-effectiveness varied based on the treatment approach and patient characteristics (Table 1). Proton therapy was more cost-effective for left-sided compared with right-sided cases, primarily due to greater reductions in CVD risk. Furthermore, incorporating RNI lowered the ICERs, due to greater reductions in overall complications. Additionally, patients at higher risk of cardiopulmonary complications—those with higher baseline CVD risk, current smokers, and older patients—experienced significantly lower ICERs, due to higher incremental QALYs.

### Sensitivity Analyses

The results of the deterministic sensitivity analyses are presented in eFigure in Supplement 1. Across all scenarios, the cost of proton therapy consistently emerged as a key factor for cost-effectiveness, as indicated by the largest variation in ICERs when this parameter was varied. Laterality influenced the relative importance of model parameters; for left-sided irradiation, parameters related to cardiovascular disease, particularly the excess CVD rate ratio and CVD mortality, were among the most influential factors underlying ICER variation, whereas for right-sided irradiation, parameters related to lung cancer, most notably the excess lung cancer rate ratio and lung cancer mortality, had greater influence. When RNI was included, parameters associated with contralateral breast cancer contributed more prominently to ICER variability. In addition, the use of DIBH attenuated the influence of parameters related to cardiovascular disease and lung cancer, as reflected by the narrower ICER ranges associated with these parameters compared with FB scenarios.

The results of the probabilistic sensitivity analyses are illustrated as cost-effectiveness acceptability curves in the Figure. The adoption of DIBH shifted the curves to the right, indicating reduced cost-effectiveness. This shift was more pronounced in scenarios involving left-sided irradiation and the inclusion of RNI.

**Figure.**
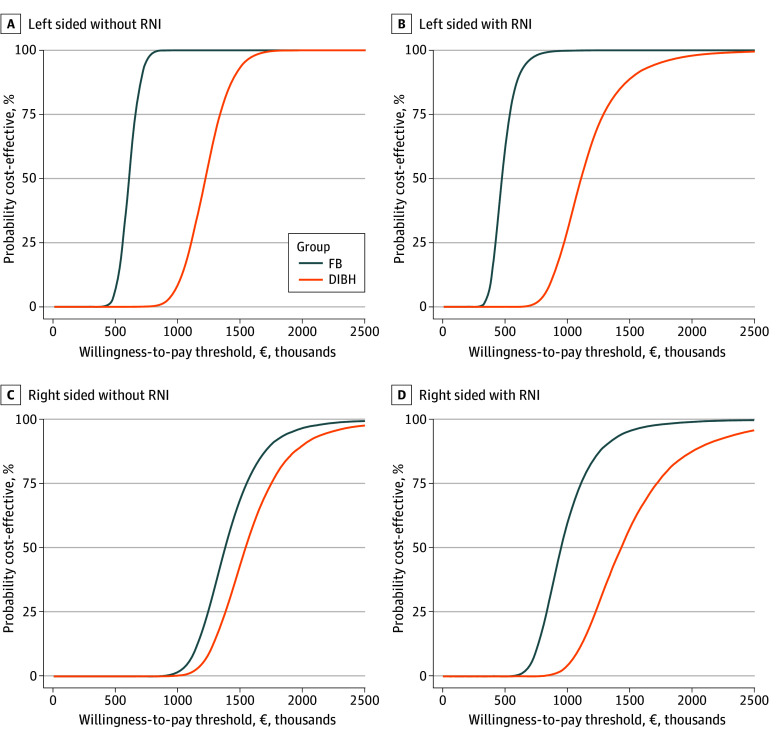
Probabilistic Sensitivity Analyses The cost-effectiveness acceptability curves illustrate the probability of proton therapy being cost-effective across various willingness-to-pay (WTP) thresholds. Each point on the curves represents the proportion of simulations from the probabilistic sensitivity analyses where proton therapy is considered cost-effective at a given WTP. These analyses were conducted using 10 000 Monte Carlo simulations to evaluate incremental costs and benefits of proton vs photon therapy across 8 clinical scenarios: left- or right-sided irradiation, with or without regional nodal irradiation (RNI), and under free breathing (FB) or deep inspiration breath-hold (DIBH). At a WTP threshold of €45 000 per quality-adjusted life-year, proton therapy was not cost-effective in any of the simulations across all scenarios.

### Scenario Analysis

Table 3 demonstrates the ICERs and the percentage of patients for whom proton therapy was deemed cost-effective across scenarios of increasing treatment capacity and progressively reduced costs.

**Table 3.** Results of Scenario Analysis^a^

Scenario and group	ICER (95% CI), €		Cost-effective, %	
	Proton vs photon FB	Proton vs photon DIBH	Proton vs photon FB	Proton vs photon DIBH
Scenario 1^b^				
Left	590 706 (504 907-710 950)	1 182 977 (904 264-1 706 656)	0.0	0.0
Left with RNI	475 768 (407 082-571 915)	1 067 379 (799 066-1 604 409)	0.0	0.0
Right	1 392 610 (985 715-2 363 810)	1 538 684 (1 038 750-2 953 698)	0.0	0.0
Right with RNI	962 524 (757 085-1 319 494)	1 385 459 (1 011 293-2 194 798)	0.0	0.0
Scenario 2^c^				
Left	305 610 (260 646-368 626)	611 263 (466 268-883 698)	0.0	0.0
Left with RNI	242 203 (206 873-242 203)	544 682 (407 141-819 970)	0.0	0.0
Right	728 765 (514 730-1 239 635)	796 618 (536 831-1 533 068)	0.0	0.0
Right with RNI	496 258 (389 782-681 271)	708 547 (516 366-1 124 246)	0.0	0.0
Scenario 3^d^				
Left	220 381 (187 625-266 288)	440 351 (335 330-637 676)	0.0	0.0
Left with RNI	172 380 (147 021-207 877)	388 423 (289 976-585 464)	1.67	0.0
Right	530 310 (373 930-903 566)	575 169 (386 784-1 108 374)	0.0	0.0
Right with RNI	356 869 (279 978-490 476)	506 186 (368 409-804 207)	0.0	0.0
Scenario 4^e^				
Left	156 493 (132 887-189 575)	312 223 (237 177-453 255)	0.0	0.0
Left with RNI	120 039 (102 155-145 073)	271 289 (202 147-409 675)	6.44	0.0
Right	381 545 (268 384-651 644)	408 943 (274 307-790 017)	0.0	0.0
Right with RNI	252 381 (197 667-347 453)	354 494 (257 498-564 301)	0.0	0.0
Scenario 5^f^				
Left	120 215 (101 805-146 015)	239 483 (181 443-348 535)	4.17	0.0
Left with RNI	90 318 (76 679-109 411)	204 776 (152 275-309 857)	11.32	0.0
Right	297 072 (208 452-508 595)	314 554 (210 438-609 244)	0.0	0.0
Right with RNI	193 050 (150 928-266 240)	268 358 (195 519-428 074)	1.67	0.0

Abbreviations: DIBH, deep inspiration breath-hold; FB, free breathing; ICER, incremental cost-effectiveness ratio; RNI, regional nodal irradiation.

^a^ The results present the ICERs and the proportion of patients for whom proton therapy is considered cost-effective under scenarios of increasing treatment capacity and progressively reduced costs. In each scenario, patients with breast cancer were assumed to constitute the largest patient group, accounting for 35% of the total patient volume. The scenarios are adapted from Chen et al.^32^ The distribution of patients with breast cancer across clinical subgroups (left, 35%; left with RNI, 17%; right, 33%; right with RNI, 15%) within the total annual breast cancer case volume were based on national incidence data. These values were used as fixed model inputs to define the case mix.

^b^ Base case: annual patients = 244; annual patients with breast cancer = 86; cost of proton therapy = €56 550.

^c^ 56% Capacity: annual patients = 450; annual patients with breast cancer = 159; cost of proton therapy = €32 703.

^d^ 75% Capacity: annual patients = 600; annual patients with breast cancer = 211; cost of proton therapy = €25 574.

^e^ Maximum capacity: annual patients = 800; annual patients with breast cancer = 282; cost of proton therapy = €20 230.

^f^ Maximum capacity with 15% cost reduction: annual patients = 800; annual breast cancer patients = 282; cost of proton therapy = €17 196.

At 75% treatment capacity, proton therapy was cost-effective compared with photon FB for 1 of 211 annual patients with breast cancer (0.3%), specifically current smokers aged 70 to 79 years receiving left-sided irradiation with RNI. At full capacity, this increased to 3 of 282 annual patients (1.1%), extending to current smokers aged 60 to 79 years receiving left-sided irradiation with RNI. With full treatment capacity and a 15% cost reduction, proton therapy was cost-effective for 10 of 282 annual patients (3.6%), including (1) individuals at high baseline cardiopulmonary risk or current smokers aged 70 to 79 years receiving left-sided irradiation with FB, (2) patients aged 60 to 79 years receiving left-sided irradiation with RNI, and (3) current smokers aged 70 to 79 years receiving right-sided irradiation with RNI.

With full capacity and a 15% cost reduction, proton therapy was cost-effective for X of Y patients (3.6%), including: (1) high-risk individuals or current smokers aged 70 to 79 years receiving left breast irradiation only (data), (2) those aged 60 to 79 years undergoing left breast irradiation with RNI (data), and (3) current smokers aged 70 to 79 years receiving right breast irradiation with RNI (data).

## Discussion

While this economic evaluation found that proton therapy improved QALYs, the findings also suggests that its high cost limits its cost-effectiveness to a minority of patients with breast cancer in Belgium. Consistent with previous studies, our findings indicate that proton therapy is more likely to be cost-effective in patients with a higher baseline CVD risk.^9,10,14,15^ Our work also extends prior research by emphasizing the importance of smoking status. Smokers, who are at increased risk for both CVD and lung cancer, may particularly benefit from proton therapy’s capability to spare the heart and lungs. This aspect was likely not identified in previous studies because they did not include lung cancer in their models or assumed that the risks of lung cancer and CVD were independent of smoking status. Additionally, similar to previous studies, our analysis indicates that proton therapy is more likely to be cost-effective in scenarios where photon-based treatments deliver higher MHDs, particularly in left-sided irradiation involving RNI.^14,15^ Our study extends this observation by showing greater benefits in the context of RNI, not only due to the reduced cardiovascular risks but also because of decreased pulmonary and contralateral breast cancer risks.

In contrast with other studies, the ICERs in the base case of this analysis were higher, primarily due to the higher estimated costs of proton therapy. This study used patient-level cost data from the Chen et al^32^ analysis of patients with breast cancer treated at a proton center, while previous studies relied on theoretical estimates^9,10^ or cost data from other tumor types.^14,15,16^ There is a significant gap in high-quality cost data for proton therapy in breast cancer, and the work of Chen et al^32^ suggests that the costs in those studies may have been underestimated. Furthermore, the base-case cost in this study reflects the start-up phase of a proton therapy center, whereas previous studies did not account for cost differences related to varying capacity levels. This distinction is critical because achieving maximum capacity is not guaranteed. Even under the most optimistic cost scenario in this study—assuming maximum capacity and a 15% cost reduction (eg, from technological advancements)—proton therapy was cost-effective for only 3.6% of patients.

This small proportion raises concerns about the feasibility of implementing proton therapy at scale. In the Netherlands, capacity targets were not met, and challenges were identified for both patients and oncologists.^34^ Ensuring adequate capacity may require long-distance travel, adding logistical and financial burdens that increase overall costs.^35^ Greater travel distances have been associated with higher mastectomy rates and can result in poorer outcomes due to lower radiotherapy adherence.^36,37,38^ Additionally, identifying eligible patients adds complexity, increasing oncologists’ workload and potentially delaying treatment. Low patient volumes also threaten financial viability, as seen in the US, where several proton centers faced bankruptcy due to insufficient demand.^39,40^

A more pressing concern for the future of proton therapy in breast cancer treatment is that the arguments in its favor have weakened over time. At the start of the 21st century, the outlook appeared promising, driven by apprehensions about cardiotoxicity with photon therapy and the expectation of decreasing costs for proton therapy.^10,41^ However, 2 decades later, the anticipated reduction in proton therapy’s cost has not materialized, and substantial cost savings appear unlikely in the foreseeable future.^42^ Moreover, the development more affordable alternatives like DIBH has further weakened the case for proton therapy. In this study, proton therapy was not cost-effective for any patients when compared with photon DIBH. This is because proton therapy’s main theoretical advantages—reduced cardiopulmonary toxicity—were significantly diminished when compared with photon DIBH, which also spares the heart and lungs. Additionally, systematic reviews indicate that advancements in photon therapy in general have significantly reduced doses to critical organs such as the heart,^12,13^ lungs,^43^ and contralateral breast,^44^ thereby diminishing the advantages that proton therapy once offered. This is especially notable for MHDs, which have historically been the Achilles heel of photon therapy. A systematic review^12^ reported an average MHD of just 2.6 Gy for the most recent year included (2017), underscoring the significant advancemens.

Moreover, it remains uncertain whether the dosimetric advantages of proton therapy translate into meaningful clinical benefits. To date, no randomized clinical trials for breast cancer have been published,^45^ and although several are under way not all consistently include photon DIBH as comparator, potentially overstating proton therapy’s benefits.^46,47,48^ Adding to this uncertainty is the clinical evidence suggesting increased dermatological toxic effects with proton therapy.^45^ The traditional passive scattering technique is associated with higher skin doses, raising concerns about its cosmetic impacts.^49^ Although the introduction of pencil beam scanning has reduced skin doses, evidence still suggests higher rates of radiation dermatitis.^50^ Moreover, there may be long-term consequences. In a prospective trial a higher incidence of skin toxicities was observed even after 7 years.^51^ Given the importance of cosmetic outcomes for long-term well-being, these potential negative effects may undermine the cost-effectiveness of proton therapy.^52,53^

Photon DIBH, in contrast, is generally well-tolerated, less costly, and—despite a modest increase in treatment time—can be integrated into existing clinical workflows, making it a more scalable approach.^17^ Furthermore, DIBH is endorsed by leading professional organizations, including the American Society for Radiation Oncology^23^ and the European Society for Radiotherapy and Oncology,^54^ reflecting its growing integration into standard clinical practice.

Clinical decision-making should ultimately be guided by observed patient outcomes. However, the long latency of key radiation-induced toxic effects means that definitive evidence on the long-term clinical benefits of proton therapy in this population may take years to emerge. While modeling studies are not practice-changing in isolation, they can critically appraise current practice patterns. The global uptake of proton therapy in breast cancer has often preceded robust long-term evidence and occurred despite substantially higher costs. This study’s findings underscore the need for judicious patient selection and call into question the appropriateness of widespread, nonselective adoption in resource-constrained health care systems.

### Limitations

This study has limitations. Cost-effectiveness analyses are highly dependent on the context in which they are conducted, and the results of this study may therefore not be directly applicable to other countries. For example, real-world treatment costs can vary substantially across regions and institutions, which may affect the generalizability of our cost-effectiveness estimates. Such variability should be carefully considered when interpreting and applying these findings across different health care contexts. Additionally, given that Belgium has a relatively low CVD risk, proton therapy might be cost-effective for a larger proportion of patients in countries with higher baseline CVD risk. Proton therapy could also offer greater benefits for patients with a genetic predisposition to contralateral breast cancer.^55^ However, due to the small proportion of familial cases, this factor was not incorporated into the present analysis and should be examined in future research. Moreover, while the NTCP models employed in this study are based on the most robust and widely accepted evidence derived from large-scale patient cohorts, ongoing research continues to refine these models. For end points such as CVD and lung cancer, linear dose–response relationships currently provide the best fit to empirical data; however, biological responses to radiation may exhibit greater complexity. Investigations into more nuanced nonlinear dose–response relationships and the incorporation of additional patient-specific risk factors have the potential to improve model accuracy. As more advanced and validated models become available, future cost-effectiveness analyses should consider their integration to enhance precision.

## Conclusions

In this economic evaluation of proton therapy for breast cancer, proton therapy offered potential clinical benefits, particularly for patients at high risk of cardiopulmonary complications and in scenarios where photon therapy delivered relatively high doses to critical organs at risk, such as in left-sided breast irradiation with RNI. However, its high cost limited cost-effectiveness to a small proportion of patients. Moreover, the advantages of proton therapy were substantially diminished when compared with cardiac-sparing photon therapies, such as DIBH. These findings underscored the importance of carefully considering the selection of comparators when evaluating the cost-effectiveness of proton therapy for breast cancer.

## Notes

Supplement 1. eTable 2. Age distribution of breast cancer incidence among Belgian women eTable 1. Dosimetric data eTable 3. Cardiovascular risk factors eTable 7. Age-specific one year risk of a secondary CVD event eTable 6. Age-specific probabilities of mortality after a CVD event eTable 4. Distribution of smoking status eTable 5. Lung cancer risk factors eTable 8. Cancer mortality eTable 9. Background mortality eAppendix 1. Normal tissue compication probability models eTable 10: Age-specific baseline utilities eTable 11. Disutilities for complications eAppendix 2. Cost calculation eTable 12. Treatment costs eTable 13. Costs of complications eAppendix 3. Sensitivity analysis eFigure. Deterministic sensitivity analyses eReferences.

Supplement 2. Data Sharing Statement
