# Supplementary material for: Cost-Effectiveness of Proton Therapy Compared With Photon Therapy in Breast Cancer
Source: JAMA Netw Open. 2026 Jan 22;9(1):e2554888. doi: 10.1001/jamanetworkopen.2025.54888 (PMC12828626; doi:10.1001/jamanetworkopen.2025.54888)
Supplement: Supplement 1. — eTable 2. Age distribution of breast cancer incidence among Belgian women eTable 1. Dosimetric data eTable 3. Cardiovascular risk factors eTable 7. Age-specific one year risk of a secondary CVD event eTable 6. Age-specific probabilities of mortality after a CVD event eTable 4. Distribution of smoking status eTable 5. Lung cancer risk factors eTable 8. Cancer mortality eTable 9. Background mortality eAppendix 1. Normal tissue compication probability models eTable 10: Age-specific baseline utilities eTable 11. Disutilities for complications eAppendix 2. Cost calculation eTable 12. Treatment costs eTable 13. Costs of complications eAppendix 3. Sensitivity analysis eFigure. Deterministic sensitivity analyses eReferences. [file jamanetwopen-e2554888-s001.pdf]

Supplemental Online Content

Busschaert SL, Kimpe E, Gevaert T, De Ridder M, Putman K. Cost-effectiveness of proton therapy compared with photon therapy in breast cancer. *JAMA Netw. Open*. 2026;9(1): e2554888. doi:10.1001/jamanetworkopen.2025.54888

eTable 1. Age distribution of breast cancer incidence among Belgian women

eTable 2. Dosimetric data

eTable 3. Cardiovascular risk factors

eTable 4. Age-specific one year risk of a secondary CVD event

eTable 5. Age-specific probabilities of mortality after a CVD event

eTable 6. Distribution of smoking status

eTable 7. Lung cancer risk factors

eTable 8. Cancer mortality

eTable 9. Background mortality

eAppendix 1. Normal tissue compication probability models

eTable 10. Age-specific baseline utilities

eTable 11. Disutilities for complications

eAppendix 2. Cost calculation

eTable 12. Treatment costs

eTable 13. Costs of complications

eAppendix 3. Sensitivity analysis

eFigure. Deterministic sensitivity analyses

eReferences.

This supplemental material has been provided by the authors to give readers additional information about their work.

© 2026 Busschaert S-L et al. *JAMA Network Open*.

eAppendix 1. Normal tissue complication probability models

1.1. Cardiovascular disease

The SCORE2 algorithms[1-3] were applied to estimate the 10-year total (fatal and non-fatal) risk of first-onset cardiovascular disease (CVD) before radiotherapy, referred to as baseline total CVD risk. These algorithms require the following input parameters: age, sex, total cholesterol (mmol/L), HDL cholesterol (mmol/L), systolic blood pressure (mmHg), presence of diabetes, and smoking status (current smoker or not). Age was derived from the age distribution of female breast cancer incidence in Belgium (eTable 1), while the other variables were obtained from the Belgian Health Examination Survey—a cross-sectional epidemiological study collecting objective health data from a representative sample of the Belgian population (eTable 3)[4]. For diabetic individuals, additional parameters are included to refine risk estimation: age at diabetes diagnosis, glycated hemoglobin (HbA1c, mmol/mol), and creatinine-based estimated glomerular filtration rate (eGFR, mL/min/1.73 m^2). These data were sourced from the most recent IQED (Initiative for Quality Improvement and Epidemiology in Diabetes) audit, a large-scale periodic study assessing diabetes care quality and gathering comprehensive data on Belgian individuals with diabetes[5] (eTable 3).

The SCORE2 algorithms are applied by first calculating a linear predictor using patient-specific inputs, including age, sex, total cholesterol, HDL cholesterol, systolic blood pressure, diabetes status, and smoking status. This linear predictor is then transformed into a predicted 10-year total CVD risk. To enhance accuracy, the predicted risk is recalibrated based on regional scale coefficients, categorizing the individual into one of four risk levels: low, moderate, high, or very high. Below is an example illustrating this process for a non-diabetic female residing in a low-risk region, such as Belgium.

© 2026 Busschaert S-L et al. *JAMA Network Open*.

$$Linear\ predictor = 0.4648 * \left(\frac{Age - 60}{5}\right) + \left(0.1002 * \left(\frac{TotalCholesterol - 6}{1}\right)\right) + \left(-0.2606 * \left(\frac{HDLCholesterol - 1.3}{0.5}\right)\right) + \left(0.3131 * \left(\frac{SystolicBloodPressure - 120}{20}\right)\right) + (0.8096 * Diabetes) + (0.7744 * Smoking) + \left(-0.0226 * \left(\frac{Age - 60}{5}\right) * (TotalCholesterol - 6)\right) + \left(0.0613 * \left(\frac{Age - 60}{5}\right) * \left(\frac{HDLCholesterol - 1.3}{0.5}\right)\right) + \left(-0.0277 * \left(\frac{Age - 60}{5}\right) * \left(\frac{SystolicBloodPressure - 120}{20}\right)\right) + \left(-0.1272 * \left(\frac{Age - 60}{5}\right) * Diabetes\right) + \left(-0.1088 * \left(\frac{Age - 60}{5}\right) * Smoking\right)$$

$$Predicted\ risk = 1 - 0.9776^{EXP(linear\ predictor)}$$

$$Baseline\ risk = 1 - EXP\left(-EXP\left(-0.738 + 0.7019 * LN\left(-LN(1 - Predicted\ risk)\right)\right)\right)$$

Based on the well-established dose-response relationship between mean heart dose (MHD) and CVD risk, an excess CVD rate ratio of 7.4% (95% C.I. [2.9% - 14.5%]) per Gy[6] was used to accommodate the cardiotoxic effects of radiation. The post-radiotherapy risk was calculated using the following equation:

$$Post - Radiotherapy\ risk = Baseline\ risk\ (1 + (MHD * 0.074))$$

1.2. Lung cancer

Baseline 6-year lung cancer risks were estimated using the PLCOall2014 model, which provides distinct equations for current smokers, former smokers, and never smokers. The age-specific distribution of smoking status is outlined in eTable 6, while predictor values (excluding age, detailed in eTable 1) are provided in eTable 7. The PLCOall2014 model incorporates the following predictors

© 2026 Busschaert S-L et al. *JAMA Network Open*.

for all individuals: age, education level, body mass index (BMI), chronic obstructive pulmonary disease, the presence of emphysema or chronic bronchitis, personal history of cancer (present in all patients), family history of lung cancer, and race/ethnicity. For current and former smokers, the average number of cigarettes smoked per day and smoking duration are also included, with time since quitting additionally accounted for in former smokers. Below is an example calculation for a smoker, with ethnicity set to the default value (White):

$$
\begin{aligned}
Baseline\ Risk = EXP(&(Age - 62) * 0.079597 \\
&+ (Education - 4) * -0.0879289 \\
&+ (BMI - 27) * -0.028948 \\
&+ ChronicPulmonaryDisease * 0.3457265 \\
&+ FamilyHistoryLungCancer * 0.5856777 \\
&+ PersonalHistoryCancer * \ 0.4845208 \\
&+ 2.799727 \\
&+ ((\frac{CigarettesSmoked}{100})^{-1} - 4.021541613) * \ -0.1815486 \\
&+ (YearsSmoked - 27) * 0.0305566 \\
&- 7.02198)
\end{aligned}
$$

Drawing on the relationship between bilateral mean lung dose (MLD) and lung cancer risk observed in a meta-analysis of randomised controlled trials in modern breast cancer radiotherapy,[7] an excess lung cancer rate ratio of 11% (95% C.I. [5% - 20%]) per Gy[7] was applied to account for the iatrogenic pulmonary impact of RT. Post-radiotherapy lung cancer risks are calculated with the following equation:

$$Post - Radiotherapy\ risk = \ Baseline\ risk\ (1 + (MLD * 0.11))$$

1.3. Contralateral breast cancer

The Schneider model was employed to estimate the probabilities of developing contralateral breast cancer following proton and photon radiotherapy[8]. The model calculates the Excess Absolute Risk

© 2026 Busschaert S-L et al. *JAMA Network Open*.

(EAR) in a small volume element of an organ, factoring in dose-response and patient-specific age effects. The EAR is expressed as:

$$EAR_{D,age_x,age_a} = \beta * RED(D) * \mu_{age_x,age_a}$$

Here, β represents the initial slope of the dose-response relationship at low doses, and $RED(D)$ (Risk Equivalent Dose) is a function of the dose that describes the risk for radiation-induced cancer. In this model, β_{EAR} is set to 8.2 (in units of 10,000 person-years per Gy).

The modifying function μ_{age_x,age_a} adjusts for patient age at exposure (age_x) and age at risk (age_a), as shown by:

$$\mu_{age_x,age_a} = \exp\left(\gamma_e(age_x - 30) + \gamma_a \ln\left(\frac{age_a}{70}\right)\right)$$

where $\gamma_e = -0.037$ (the age at exposure modifier) and $\gamma_a = 1.7$ (the age attained modifier).

A mechanistic model incorporating cell killing and fractionation effects was applied to calculate the risk equivalent dose for a given dose D:

$$RED(D) = \frac{e^{-\propto D}}{\propto R}(1 - 2R + R^2 e^{\propto D} - (1 - R)^2 e^{\frac{-\propto D}{1-R}}$$

where R is the repair/repopulation parameter, ranging from 0 (no repair) to 1 (full repair). The parameters α and β are taken from the linear-quadratic model, which models the radiation response, with α adjusted to account for fractionation as:

$$\alpha' = \alpha + \beta \frac{d}{D_T} d_T$$

where D_T is the total prescribed dose and d_T is the dose per fraction.

By applying this model, this study quantified contralateral breast cancer risk due to photon and proton radiotherapy, incorporating dose distribution and fractionation patterns unique to each radiation type, as well as age-related factors specific to each patient.

© 2026 Busschaert S-L et al. *JAMA Network Open*.

1.4. Radiation pneumonitis

In this study, the Mean Lung Dose (MLD) version of the Lyman-Kutcher-Burman (LKB) model was used to estimate the probability of developing radiation pneumonitis after radiotherapy. The LKB model is widely used for predicting the likelihood of normal tissue complications by modelling dose-response relationships for specific organs, in this case, lung tissue[9]. The NTCP model integrates dose, tissue sensitivity, and response slope to estimate individual risk levels for radiation pneumonitis, allowing for patient-specific risk assessment based on MLD. The probability of radiation pneumonitis or NTCP, is calculated as:

$$NTCP = \Phi\left(\frac{MLD - D_{50}}{m * D_{50}}\right)$$

where:

Φ: Phi represents the cumulative distribution function of the standard normal distribution, which converts the calculated risk factor into a probability, providing a continuous measure of the likelihood of developing radiation pneumonitis.

D_{50}: The parameter D_{50} indicates the mean lung dose at which there is a 50% probability of radiation pneumonitis. It reflects the lung tissue's sensitivity to radiation, where a higher D_{50} suggests greater resistance to radiation-induced damage.

m: The steepness parameter m controls the slope of the dose-response curve around D_{50}, indicating how sharply the probability of pneumonitis increases as the mean lung dose approaches D_{50}. A higher m value corresponds to a steeper dose-response relationship, reflecting a more rapid increase in risk around the D_{50} threshold.

Research by Seppenwoolde et al.[10] comparing various NTCP models for predicting radiation pneumonitis suggests that the relationship between MLD and NTCP can be characterized with a D_{50} of 30.8 Gy and a steepness parameter m of 0.37. These values were therefore adopted in the analysis.

© 2026 Busschaert S-L et al. *JAMA Network Open*.

eAppendix 2. Cost calculation

Treatment costs for photon breast irradiation with FB and DIBH were derived from Belgian time-driven activity-based costing (TD-ABC) analyses, while proton therapy costs were obtained from a Dutch TD-ABC analysis. The Netherlands was selected as a suitable alternative, given its proximity, comparable healthcare system, and the relevance of its data. Additionally, the Dutch cost data is recent, reflecting current practices and providing a reliable basis for comparison. Importantly, all costs were determined using the same TD-ABC methodology, a robust approach for calculating detailed, patient-specific costs, particularly effective in capturing the complexities of RT costs.

With respect to the costs associated with RT complications, only direct healthcare costs were considered, and all cost estimates were derived from Belgian data, ensuring compliance with the guidelines set forth by the Belgian Healthcare Knowledge Centre. To ensure consistency, all costs were adjusted to 2024 euros, with further details provided in eTable 12. For CVD, the included costs encompass hospitalization, coronary revascularization procedures, and follow-up care, which includes rehabilitation and outpatient resources such as tests, imaging, and prescribed medications[11]. The costs differ between fatal and non-fatal cases and are further divided into short-term and long-term costs for non-fatal cases. Direct healthcare costs for lung and breast cancer reflect total healthcare spending, covering both patient out-of-pocket payments and reimbursed costs. These include hospital stays, hospital-based and outpatient care, and reimbursed prescription drugs[12] Costs associated with radiation pneumonitis are based on Belgian treatment guidelines, which recommend a corticosteroid regimen[13]. The cost of corticosteroids was obtained from the Belgisch Centrum voor Farmacotherapeutische Informatie (BCFI).

© 2026 Busschaert S-L et al. *JAMA Network Open*.

eAppendix 3. Sensitivity analysis

To evaluate the robustness of the model results, both deterministic and probabilistic sensitivity analyses (DSA and PSA, respectively) were conducted, following ISPOR-SMDM (International Society for Pharmacoeconomics and Outcomes Research-Society for Medical Decision Making) best practice recommendations.[14]

DSAs were performed for each of the eight scenarios (i.e., left- or right-sided breast radiotherapy, with or without RNI, comparing proton therapy to Photon FB or Photon DIBH) to assess the impact of key parameters on the incremental cost-effectiveness ratios (ICERs). Individual model inputs were varied within their 95% confidence intervals based on values reported in the literature. When confidence intervals were unavailable, parameters were adjusted by ±30% from the base case estimates. Results of the DSA were presented using tornado diagrams to identify the most influential parameters driving model outcomes. The sensitivity analysis focused on the parameters detailed in the tables included in this appendix. Key aspects included:

- Cost inputs: Variations in the costs of photon and proton therapy, as well as costs associated with complications such as cardiovascular disease, secondary malignancies, and radiation pneumonitis.
- Utility values: Modifications to baseline utilities and the disutilities linked to treatment-related complications.
- Clinical probability factors: Adjustments to parameters driving risk models for complications (e.g., cholesterol levels for cardiovascular disease risk, daily cigarette consumption for lung cancer risk), along with changes in mortality risks associated with these complications.

To capture the overall uncertainty in the model, probabilistic sensitivity analyses were performed by simultaneously varying all input parameters. The PSA was conducted using 10,000 Monte Carlo simulations for each of the eight scenarios (i.e., left- or right-sided breast radiotherapy, with or without RNI, comparing proton therapy to photon FB or photon DIBH). This approach generated distributions of ICERs, summarised using cost-effectiveness planes and cost-effectiveness acceptability curves. These outputs provided insights into the probability of proton therapy being cost-effective at varying

willingness-to-pay (WTP) thresholds. Parameters were varied according to the distributions outlined in the tables of this Appendix. For instance, gamma distributions were applied to account for the positive skew of costs and beta distributions were used to reflect the bounded nature0of utilities between 0 and 1. Lognormal distributions were applied to the excess CVD rate ratio of 7.4% (95% CI: 2.9%–14.5%) per Gy and the excess lung cancer rate ratio of 11% (95% CI: 5%–20%) per Gy.

© 2026 Busschaert S-L et al. *JAMA Network Open*.

eFigure. Deterministic sensitivity analyses

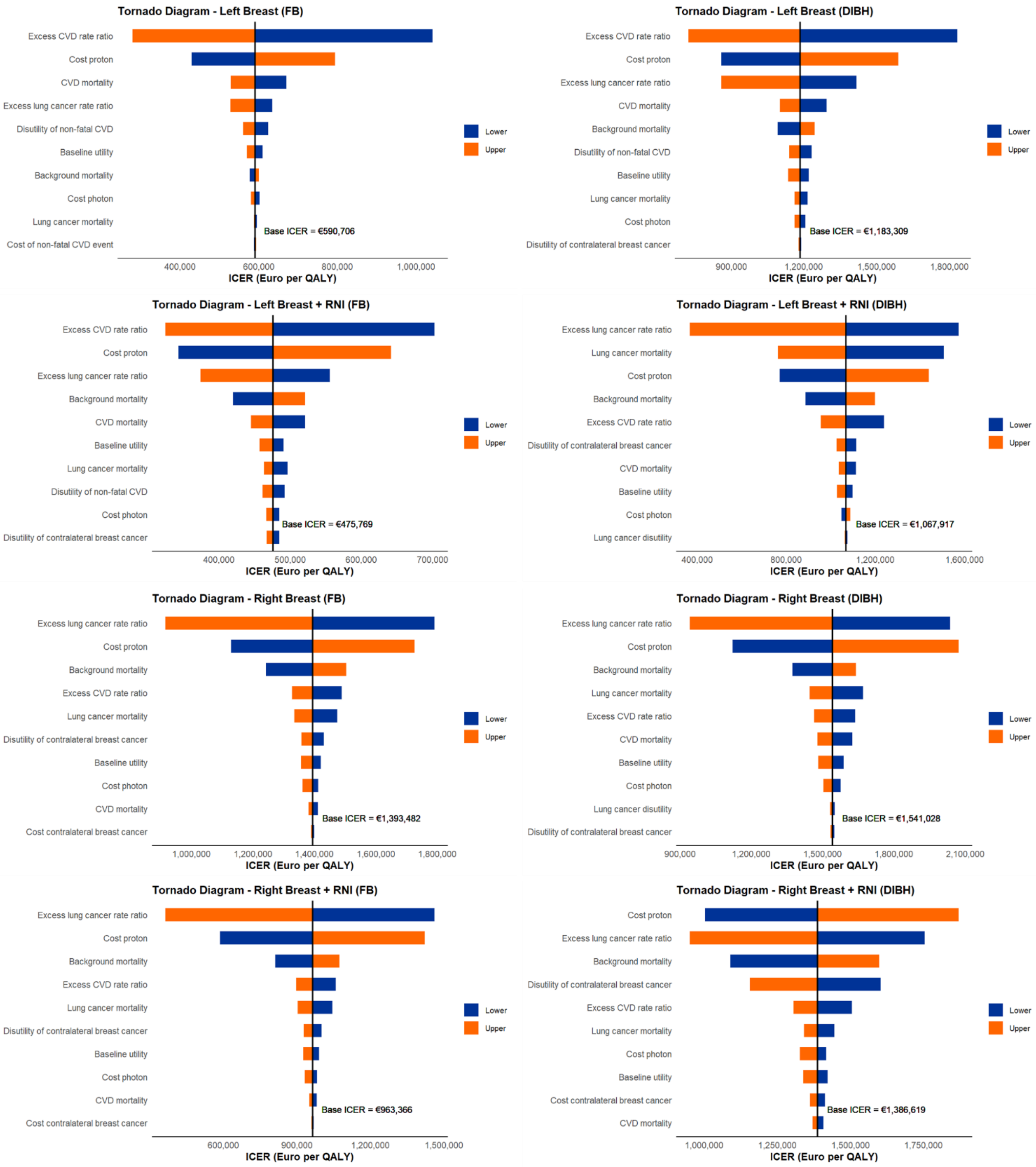

The tornado diagrams illustrate the influence of various parameters on the mean incremental cost-effectiveness ratio (ICER), focusing on the 10 most impactful parameters for each scenario. Parameter values were varied within their 95% confidence intervals where available or by ±30% of the base case values when confidence intervals were not reported. Detailed descriptions of base case values and the corresponding ranges for all parameters are provided in the Appendix

eTable 2. Dosimetric data

**Photon FB**[15-19]	**Mean Heart Dose**	**Bilateral Mean Lung Dose**	**Mean Contralateral Breast Dose**
Left-sided without RNI	3.89 Gy (± 2.13)	3.00 Gy (± 1.51)	0.52 Gy (± 0.16)
Left-sided with RNI	4.77 Gy (± 2.39)	9.18 Gy (± 1.30)	1.45 Gy (± 0.80)
Right-sided without RNI	0.40 Gy (± 0.14)	4.25 Gy (± 1.41)	0.52 Gy (± 0.16)
Right-sided with RNI	1.01 Gy (± 0.61)	8.58 Gy (± 1.82)	1.45 Gy (± 0.80)
**Photon DIBH**[15-19]	**Mean Heart Dose**	**Bilateral Mean Lung Dose**	**Mean Contralateral Breast Dose**
Left-sided without RNI	1.68 Gy (± 1.02)	2.75 Gy (± 1.24)	0.52 Gy (± 0.16)
Left-sided with RNI	1.93 Gy (± 1.06)	7.85 Gy (± 0.77)	1.45 Gy (± 0.80)
Right-sided without RNI	0.39 Gy (± 0.12)	3.80 Gy (± 1.20)	0.52 Gy (± 0.16)
Right-sided with RNI	0.80 Gy (± 0.32)	7.17 Gy (± 1.44)	1.45 Gy (± 0.80)
**Proton**[17-21]	**Mean Heart Dose**	**Bilateral Mean Lung Dose**	**Mean Contralateral Breast Dose**
Left-sided without RNI	0.40 Gy (± 0.12)	1.40 Gy (± 0.30)	0.40 Gy (± 0.12)
Left-sided with RNI	0.88 Gy (± 0.29)	4.20 Gy (± 1.10)	0.20 Gy (± 0.10)
Right-sided without RNI	0.20 Gy (± 0.10)	1.40 Gy (± 0.30)	0.40 Gy (± 0.12)
Right-sided with RNI	0.41 Gy (± 0.21)	4.20 Gy (± 1.10)	0.20 Gy (± 0.10)

DIBH: deep inspiration breath hold; **FB:** free breathing; **RNI:** regional nodal irradiation

This table presents the dosimetric data for each treatment approach. A systematic review of the academic literature was conducted to obtain representative dosimetric data for left- or right-sided irradiation with or without RNI using proton therapy, photon FB, or photon DIBH. The search prioritised recent studies to ensure the data reflected current practices and aimed to maximise reliability by focusing on well-designed, peer-reviewed research.

© 2026 Busschaert S-L et al. *JAMA Network Open*.

eTable 1. Age distribution of breast cancer incidence among Belgian women

**Age group**	**Proportion**
40-44 yrs.	6.55%
45-49 yrs.	10.58%
50-54 yrs.	12.52%
55-59 yrs.	13.87%
60-64 yrs.	15.46%
65-69 yrs.	16.03%
70-74 yrs.	13.72%
75-79 yrs.	11.27%

The data provide the proportion of patients in each age group and were obtained from the Belgian cancer registry.

© 2026 Busschaert S-L et al. *JAMA Network Open*.

eTable 3. Cardiovascular risk factors

Risk factor	Base case value [95% C.I.]	Distribution
Blood Pressure (in mmHg)		
40-64 yrs.	120.50 [118.9 – 122.1]	Normal
>64 yrs.	135.30 [132.5 – 138.4]	Normal
Total cholesterol (in mmol/l)		
40-64 yrs.	5.12 [5.03 – 5.22]	Normal
>64 yrs.	5.10 [4.87 – 5.34]	Normal
HDL cholesterol (in mmol/l)		
40-64 yrs.	1.49 [1.45 – 1.53]	Normal
>64 yrs.	1.63 [1.56 – 1.71]	Normal
Diabetes (proportion)		
40-64 yrs.	0.05 [0.03 – 0.07]	Beta
>64 yrs.	0.13 [0.05 – 0.15]	Beta
Smokers (proportion)		
40-44 yrs.	0.12 [0.08 – 0.15]	Beta
45-49 yrs.	0.18 [0.13 – 0.24]	Beta
50-54 yrs.	0.16 [0.11 – 0.21]	Beta
55-59 yrs.	0.15 [0.11 – 0.20]	Beta
60-64 yrs.	0.20 [0.14 – 0.26]	Beta
65-69 yrs.	0.10 [0.07 – 0.14]	Beta
70-74 yrs.	0.09 [0.06 – 0.12]	Beta
75-79 yrs.	0.04 [0.03 – 0.05]	Beta
Duration of diabetes (in yrs.)		
40-49 yrs.	6.00 [5.90 – 6.10]	Normal
50-59 yrs.	10.00 [9.97 – 10.03]	Normal
60-69 yrs.	16.00 [15.95 – 16.05]	Normal

© 2026 Busschaert S-L et al. *JAMA Network Open*.

70-79 yrs.	26.00 [25.94 – 26.06]	Normal

**Risk factor**	**Base case value [95% C.I.]**	**Distribution**
HbA1c (in mmol/mol)		
<50 yrs.	62.86 [42.09 – 83.63]	Normal
50-75 yrs.	59.48 [44.18 – 74.78]	Normal
>75 yrs.	56.20 [44.18 – 68.22]	Normal
eGFR (in ml/min/1.73m^2)	66.49 [46.54 – 86.44]	Normal

The given cardiovascular risk factors were used to calculate total CVD risk using the SCORE2 algorithms and were obtained from the Belgium Health Examination Survey[4]. Additional parameters for diabetic individuals were sourced from the 2022 IQED audit (i.e., duration of diabetes, HbA1c, and eGFR.

© 2026 Busschaert S-L et al. *JAMA Network Open*.

eTable 6. Distribution of smoking status

Age group	Current	Former	Never
40-44 yrs.	11.52%	18.93%	69.55%
45-49 yrs.	18.43%	15.68%	65.99%
50-54 yrs.	16.31%	22.08%	61.61%
55-59 yrs.	15.26%	27.84%	56.91%
60-64 yrs.	20.12%	32.30%	47.57%
65-69 yrs.	10.40%	29.80%	59.80%
70-74 yrs.	9.10%	26.59%	64.31%
75-79 yrs.	3.71%	15.53%	80.76%

The data present the age-specific distribution of smoking status among Belgian women and were obtained from the Belgian Health Examination Survey[4].

© 2026 Busschaert S-L et al. *JAMA Network Open*.

eTable 7. Lung cancer risk factors

Risk factor	Base case value [95% C.I.]	Distribution
Education level (mean)		
All patients	3.18 [2.23 - 4.13]	Normal
Body Mass Index (mean, in kg/m^2)		
40-44 yrs.	25.60 [24.60 - 26.60]	Normal
45-49 yrs.	25.60 [25.00 - 26.20]	Normal
50-54 yrs.	25.50 [24.90 - 26.00]	Normal
55-59 yrs.	26.20 [25.50 - 27.00]	Normal
60-64 yrs.	26.00 [25.30 - 26.70]	Normal
65-69 yrs.	26.10 [25.40 - 26.80]	Normal
70-74 yrs.	26.60 [25.90 - 27.30]	Normal
75+ yrs.	26.10 [25.30 - 26.80]	Normal
COPD, emphysema or chronic bronchitis (probability)		
40-44 yrs.	2.70 [0.50 - 4.80]	Beta
45-49 yrs.	2.60 [1.10 - 4.10]	Beta
50-54 yrs.	4.30 [2.30 - 6.40]	Beta
55-59 yrs.	4.70 [1.90 - 7.40]	Beta
60-64 yrs.	7.00 [4.20 - 9.80]	Beta
65-69 yrs.	5.70 [2.00 - 9.40]	Beta
70-74 yrs.	6.00 [2.90 - 9.20]	Beta
75+ yrs.	7.70 [3.00 - 12.40]	Beta
Family history of lung cancer (probability)		
All patients	0.24 [0.17 - 0.31]	Beta
Number of cigarettes smoked per day (mean)		
40-44 yrs.	16.70 [13.10 - 20.30]	Normal
45-49 yrs.	14.10 [12.10 - 16.10]	Normal

© 2026 Busschaert S-L et al. *JAMA Network Open*.

50-54 yrs.	15.30 [13.10 - 17.50]	Normal

**Risk factor**	**Base case value [95% C.I.]**	**Distribution**
Number of cigarettes smoked per day (mean)		
55-59 yrs.	12.20 [9.50 - 14.80]	Normal
60-64 yrs.	14.50 [11.60 - 17.40]	Normal
65-69 yrs.	18.60 [14.00 - 23.10]	Normal
70-74 yrs.	12.10 [9.40 - 14.80]	Normal
75+ yrs.	18.20 [9.60 - 26.80]	Normal
Number of years smoked, current smokers (mean)		
40-44 yrs.	23.40 [22.60 - 24.20]	Normal
45-49 yrs.	27.90 [26.60 - 29.20]	Normal
50-54 yrs.	32.10 [30.50 - 33.60]	Normal
55-59 yrs.	38.00 [37.10 - 39.00]	Normal
60-64 yrs.	43.50 [42.70 - 44.30]	Normal
65-69 yrs.	47.30 [46.30 - 48.30]	Normal
70-74 yrs.	52.10 [51.10 - 53.10]	Normal
75+ yrs.	54.00 [51.60 - 56.50]	Normal
Number of years smoked, former smokers (mean)		
40-44 yrs.	8.92 [8.62 - 9.23]	Normal
45-49 yrs.	11.15 [10.63 - 11.67]	Normal
50-54 yrs.	11.41 [10.85 - 11.98]	Normal
55-59 yrs.	14.47 [14.13 - 14.81]	Normal
60-64 yrs.	18.02 [17.69 - 18.35]	Normal
65-69 yrs.	20.86 [20.42 - 21.30]	Normal
70-74 yrs.	17.61 [17.27 - 17.94]	Normal
75+ yrs.	21.55 [20.59 - 22.50]	Normal
Number of years quit smoking, former smokers (mean)		

© 2026 Busschaert S-L et al. *JAMA Network Open*.

40-44 yrs.	14.48 [13.98 - 14.97]	Normal
45-49 yrs.	16.75 [15.97 - 17.53]	Normal
**Risk factor**	**Base case value [95% C.I.]**	**Distribution**
Number of years quit smoking, former smokers (mean)		
55-59 yrs.	23.53 [22.97 -24.09]	Normal
60-64 yrs.	25.48 [25.01 - 25.95]	Normal
65-69 yrs.	26.44 [25.88 - 27.00]	Normal
70-74 yrs.	34.49 [33.83 - 35.16]	Normal
75+ yrs.	32.45 [31.01 - 33.90]	Normal

The presented lung cancer risk factors were used to calculate 6-year lung cancer risk with the $PLCO_{all2014}$ equations. The mean education level was derived from official national statistics[22], while the likelihood of a family history of lung cancer was sourced from a recent survey conducted in Belgium[23]. The remaining factors were obtained from the Belgium Health Examination Survey[4].

© 2026 Busschaert S-L et al. *JAMA Network Open*.

eTable 5. Age-specific probabilities of mortality after a CVD event

Age group	Base case value [95% C.I.]	Distribution
45-50 yrs.	0.088 [0.062 - 0.115]	Beta
50-55 yrs.	0.125 [0.088 - 0.163]	Beta
55-60 yrs.	0.169 [0.119 - 0.220]	Beta
60-65 yrs.	0.238 [0.167 - 0.310]	Beta
65-70 yrs.	0.313 [0.219 - 0.406]	Beta
70-75 yrs.	0.400 [0.280 - 0.520]	Beta
75-80 yrs.	0.526 [0.368 - 0.684]	Beta
80-85 yrs.	0.667 [0.467 - 0.867]	Beta
>85 yrs.	0.909 [0.636 - 1.000]	Beta

Data were obtained from the papers on the SCORE2 algorithms[1,2] and give the probability of a CVD event being fatal. For example, a risk of 0.125 for women aged 50-55 years indicates that on average of 12.5% of CVD events in these patients is fatal (i.e., one in eight women decease after a CVD event). As can be derived from the table, the risk of mortality after a CVD event exhibits a pronounced dependency on age[24]. The patient's age is updated during each yearly cycle, leading to a gradual increase in the probability of a CVD event being fatal as the patient ages.

© 2026 Busschaert S-L et al. *JAMA Network Open*.

eTable 4. Age-specific one year risk of a secondary CVD event

Age group	Base case value [95% C.I.]	Distribution
≤ 50 yrs.	0.0200 [0.0140 – 0.0260]	Beta
> 50 yrs.	0.0300 [0.0210 – 0.0390]	Beta

The SCORE2 algorithms provide the total 10-year risk of first-onset CVD. After a first CVD event, patients are at high risk of further CVD events. The EUROASPIRE Risk model[25] was used to estimate the one-year risk of a recurrent fatal or non-fatal CVD event for each patient. The required inputs were acquired from the following sources: age was obtained from the patient sample, non-HDL cholesterol from the Belgian Health Examination Survey[4] and glomerular filtration rate (eGFR) from the Stockholm CREAtinine Measurements (SCREAM) project[26]. Calculations were performed with the web-based EUROASPIRE Risk Calculator[27].

© 2026 Busschaert S-L et al. *JAMA Network Open*.

eTable 8. Cancer mortality

	**Base case value [95% C.I.]**	**Distribution**
Breast cancer	6.70 [6.10 - 7.20]	Beta
Lung cancer	65.50 [64.40 - 66.50]	Beta

The table presents 5-year fatality rates for lung and breast cancer in 2022, based on data from the Belgian Cancer Registry[28].

eTable 9. Background mortality

	Base case value [95% C.I.]	Distribution
**Local breast cancer**		
<49 yrs.	0.0132 [0.0092 – 0.0171]	Beta
50-64 yrs.	0.0136 [0.0095 – 0.0176]	Beta
>64 yrs.	0.0258 [0.0181 – 0.0336]	Beta
**Regional breast cancer**		
<49 yrs.	0.0323 [0.0226 – 0.0420]	Beta
50-64 yrs.	0.0333 [0.0233 – 0.0433]	Beta
>64 yrs.	0.0634 [0.0444 – 0.0824]	Beta

The data provide 5-year risks of death from causes unrelated to CVD or lung cancer and were obtained from a population-based analysis that assessed mortality causes after breast cancer diagnosis[29]. causes of death except CVD and lung cancer are included in the risk estimates (e.g., breast cancer, Alzheimer disease, accidents).

© 2026 Busschaert S-L et al. *JAMA Network Open*.

e*Table 10. Age-specific baseline utilities*

Age group	Base case value [95% C.I.]	Distribution
40 years	0.85 [0.84 – 0.86]	Beta
41 years	0.84 [0.83 – 0.86]	Beta
42 years	0.84 [0.83 – 0.85]	Beta
43 years	0.84 [0.82 – 0.85]	Beta
44 years	0.83 [0.82 – 0.84]	Beta
45 years	0.83 [0.81 – 0.84]	Beta
46 years	0.82 [0.81 – 0.84]	Beta
47 years	0.82 [0.81 – 0.83]	Beta
48 years	0.82 [0.80 – 0.83]	Beta
49 years	0.81 [0.80 – 0.83]	Beta
50 years	0.81 [0.80 – 0.83]	Beta
51 years	0.81 [0.80 – 0.82]	Beta
52 years	0.81 [0.79 – 0.82]	Beta
53 years	0.81 [0.79 – 0.82]	Beta
54 years	0.81 [0.79 – 0.82]	Beta
55 years	0.81 [0.80 – 0.82]	Beta
56 years	0.81 [0.80 – 0.82]	Beta
57 years	0.81 [0.80 – 0.82]	Beta
58 years	0.81 [0.80 – 0.82]	Beta
59 years	0.81 [0.80 – 0.82]	Beta
60 years	0.81 [0.80 – 0.83]	Beta
61 years	0.81 [0.80 – 0.83]	Beta
62 years	0.81 [0.80 – 0.83]	Beta
63 years	0.81 [0.79 – 0.83]	Beta
64 years	0.81 [0.79 – 0.83]	Beta

© 2026 Busschaert S-L et al. *JAMA Network Open*.

65 years	0.81 [0.79 – 0.83]	Beta
**Age group**	Base case value [95% C.I.]	Distribution
66 years	0.80 [0.79 – 0.82]	Beta
67 years	0.80 [0.78 – 0.82]	Beta
68 years	0.80 [0.78 – 0.82]	Beta
69 years	0.79 [0.77 – 0.81]	Beta
70 years	0.79 [0.77 – 0.81]	Beta
71 years	0.78 [0.76 – 0.80]	Beta
72 years	0.78 [0.76 – 0.80]	Beta
73 years	0.77 [0.75 – 0.79]	Beta
74 years	0.76 [0.75 – 0.78]	Beta
75 years	0.76 [0.74 – 0.78]	Beta
76 years	0.75 [0.73 – 0.77]	Beta
77 years	0.74 [0.72 – 0.76]	Beta
78 years	0.73 [0.71 – 0.75]	Beta
79 years	0.72 [0.70 – 0.75]	Beta
80 years	0.71 [0.69 – 0.74]	Beta
66 years	0.80 [0.79 – 0.82]	Beta
67 years	0.80 [0.78 – 0.82]	Beta
68 years	0.80 [0.78 – 0.82]	Beta
69 years	0.79 [0.77 – 0.81]	Beta
70 years	0.79 [0.77 – 0.81]	Beta
71 years	0.78 [0.76 – 0.80]	Beta
72 years	0.78 [0.76 – 0.80]	Beta
73 years	0.77 [0.75 – 0.79]	Beta
74 years	0.76 [0.75 – 0.78]	Beta
75 years	0.76 [0.74 – 0.78]	Beta

© 2026 Busschaert S-L et al. *JAMA Network Open*.

76 years	0.75 [0.73 – 0.77]	Beta
77 years	0.74 [0.72 – 0.76]	Beta

**Age group**	Base case value [95% C.I.]	Distribution
78 years	0.73 [0.71 – 0.75]	Beta
79 years	0.72 [0.70 – 0.75]	Beta
80 years	0.71 [0.69 – 0.74]	Beta
81 years	0.70 [0.68 – 0.73]	Beta
82 years	0.69 [0.67 – 0.72]	Beta
83 years	0.68 [0.66 – 0.71]	Beta
84 years	0.67 [0.64 – 0.70]	Beta
85 years	0.66 [0.63 – 0.70]	Beta
86 years	0.65 [0.61 – 0.69]	Beta
87 years	0.64 [0.60 – 0.68]	Beta
88 years	0.63 [0.59 – 0.67]	Beta
89 years	0.62 [0.57 – 0.66]	Beta
90 years	0.60 [0.56 – 0.65]	Beta
91 years	0.59 [0.54 – 0.64]	Beta
92 years	0.58 [0.52 – 0.63]	Beta
93 years	0.57 [0.51 – 0.62]	Beta
94 years	0.55 [0.49 – 0.62]	Beta
95 years	0.54 [0.47 – 0.61]	Beta
96 years	0.53 [0.46 – 0.60]	Beta
97 years	0.51 [0.44 – 0.59]	Beta
98 years	0.50 [0.42 – 0.58]	Beta
99 years	0.49 [0.41 – 0.58]	Beta
100 years	0.47 [0.39 – 0.56]	Beta

The data provide the baseline utility (i.e., utility values for the "healthy" state) for patients of different ages and sexes. A 68-year-old female patient, for example, has a baseline utility of 0.8. In the

© 2026 Busschaert S-L et al. *JAMA Network Open*.

following yearly cycle, her age will increase to 69 years and her baseline utility will change to 0.79. Baseline utility values were sourced from a recent comprehensive survey of the Belgian population[30] as previous studies suggest that both breast cancer survivors and individuals of similar age from the general population demonstrate comparable overall health-related quality of life[31,32].

© 2026 Busschaert S-L et al. *JAMA Network Open*.

eTable 11. Disutilities for complications

	Base case value [95% C.I.]	Distribution
**Non-fatal CVD**		
After 0 months	-0.200 [-0.140 – -0.260]	Beta
After 6 months	-0.190 [-0.133 – -0.247]	Beta
After 12 months	-0.180 [-0.126 – -0.234]	Beta
After 18 months	-0.200 [-0.140 – -0.260]	Beta
After 24 months	-0.180 [-0.126 – -0.234]	Beta
**Lung cancer**	-0.040 [-0.028 – -0.052]	Beta
**Contralateral breast cancer**		
After 0-5 months	-0.090 [-0.063 – -0.117]	Beta
After 6-11 months	-0.060 [-0.042 – -0.078]	Beta
After 12-35 months	-0.040 [-0.028 – -0.052]	Beta
After 35-39 months	-0.020 [-0.014 – -0.026]	Beta
After ≥ 60 months	-0.010 [-0.007 – -0.013]	Beta
**Radiation pneumonitis**	-0.030 [-0.021 – -0.039]	Beta

Disutilities for complications were obtained from a recently published systematic review on health-related quality of life[33].

eTable 12. Treatment costs

	Base case value [95% C.I.]	Distribution
**Photon FB**	6,028 [5,097 – 6,959]	Gamma
**Photon DIBH**	6,645 [5,712 – 7,578]	Gamma
**Proton**	56,550 [42,998 – 73,492]	Gamma

© 2026 Busschaert S-L et al. *JAMA Network Open*.

eTable 13. Costs of complications

	Base case value [95% C.I.]	Distribution
**Non-fatal CVD**		
First year after event	31,621 [22,135 – 41,107]	Gamma
Subsequent years	8,404 [5,883 – 10,925]	Gamma
**Fatal CVD**	7,904 [5,533 – 10,275]	Gamma
**Lung cancer**	19,226 [13,458 – 24,994]	Gamma
**Breast cancer**	4,339 [3,037 – 5,641]	Gamma
**Radiation pneumonitis**	17 [12 – 22]	Gamma

The costs capture the annual direct healthcare costs per patient for each condition. All costs are expressed in 2024 euros.

© 2026 Busschaert S-L et al. *JAMA Network Open*.

eReferences

1 SCORE2 risk prediction algorithms: new models to estimate 10-year risk of cardiovascular disease in Europe. *European Heart Journal* **42**, 2439-2454 (2021).

2 SCORE2-OP risk prediction algorithms: estimating incident cardiovascular event risk in older persons in four geographical risk regions. *European Heart Journal* **42**, 2455-2467 (2021).

3 SCORE2-Diabetes: 10-year cardiovascular risk estimation in type 2 diabetes in Europe. *European heart journal* **44**, 2544-2556 (2023).

4 Nguyen, D. *et al.* The Belgian health examination survey: objectives, design and methods. *Archives of Public Health* **78**, 50 (2020).

5 A. Lavens, K. T., Prof. Dr. L. Crenier, Prof. Dr. C. De Block, Prof. Dr. C. , Mathieu, P. D. F. N., Dr. P. Oriot, Prof. Dr. J-C. Philips, Dr. M. Vandenbroucke, Dr. A. & Verhaegen, A.-S. V. INITIATIEF VOOR KWALITEITSBEVORDERING EN EPIDEMIOLOGIE BIJ DIABETES. Brussel, België : Sciensano ; 2024 Rapportnummer: D/2024/14.440/33 (2024).

6 Darby, S. C. *et al.* Risk of ischemic heart disease in women after radiotherapy for breast cancer. *New England Journal of Medicine* **368**, 987-998 (2013).

7 Taylor, C. *et al.* Estimating the risks of breast cancer radiotherapy: evidence from modern radiation doses to the lungs and heart and from previous randomized trials. *Journal of Clinical Oncology* **35**, 1641 (2017).

8 Schneider, U., Sumila, M. & Robotka, J. Site-specific dose-response relationships for cancer induction from the combined Japanese A-bomb and Hodgkin cohorts for doses relevant to radiotherapy. *Theoretical Biology and Medical Modelling* **8**, 1-21 (2011).

9 Marks, L. B. *et al.* Radiation dose–volume effects in the lung. *International Journal of Radiation Oncology* Biology* Physics* **76**, S70-S76 (2010).

10 Seppenwoolde, Y. *et al.* Comparing different NTCP models that predict the incidence of radiation pneumonitis. *International Journal of Radiation Oncology* Biology* Physics* **55**, 724-735 (2003).

11 Caekelbergh, K. *et al.* Short and long term costs associated with different cardiovascular events in Belgium. *Value in Health* **19**, A458 (2016).

12 Gorasso, V. *et al.* The incremental healthcare cost associated with cancer in Belgium: A registry-based data analysis. *Cancer Medicine* **13**, e6659 (2024).

13 Vanneste, B., Borst, G., Heuvel, M. & Belderbos, J. Diagnosis and treatment of radiation pneumonitis. (2012).

14 Briggs, A. H. *et al.* Model parameter estimation and uncertainty: a report of the ISPOR-SMDM Modeling Good Research Practices Task Force-6. *Value in Health* **15**, 835-842 (2012).

15 Busschaert, S.-L., Kimpe, E., Gevaert, T., De Ridder, M. & Putman, K. Deep Inspiration Breath Hold in Left-Sided Breast Radiotherapy: A Balance Between Side Effects and Costs. *JACC: CardioOncology* (2024).

16 Essers, M., Poortmans, P. M., Verschueren, K., Hol, S. & Cobben, D. C. Should breathing adapted radiotherapy also be applied for right-sided breast irradiation? *Acta oncologica* **55**, 460-465 (2016).

17 Aznar, M. C., Duane, F. K., Darby, S. C., Wang, Z. & Taylor, C. W. Exposure of the lungs in breast cancer radiotherapy: A systematic review of lung doses published 2010–2015. *Radiotherapy and Oncology* **126**, 148-154 (2018).

18 Paganetti, H. *et al.* The risk for developing a secondary cancer after breast radiation therapy: Comparison of photon and proton techniques. *Radiotherapy and Oncology* **149**, 212-218 (2020).

© 2026 Busschaert S-L et al. *JAMA Network Open*.

19 Ranger, A. *et al.* A dosimetric comparison of breast radiotherapy techniques to treat locoregional lymph nodes including the internal mammary chain. *Clinical Oncology* **30**, 346-353 (2018).
20 Mast, M. E. *et al.* Whole breast proton irradiation for maximal reduction of heart dose in breast cancer patients. *Breast cancer research and treatment* **148**, 33-39 (2014).
21 Taylor, C. W. *et al.* Exposure of the heart in breast cancer radiation therapy: a systematic review of heart doses published during 2003 to 2013. *International Journal of Radiation Oncology* Biology* Physics* **93**, 845-853 (2015).
22 Statbel. (2023).
23 Diab Garcia, P., Snoeckx, A., Van Meerbeeck, J. P. & Van Hal, G. A Cross-Sectional Study on the Acceptability of Implementing a Lung Cancer Screening Program in Belgium. *Cancers* **15**, 278 (2022).
24 Jørstad, H. T. *et al.* Estimated 10-year cardiovascular mortality seriously underestimates overall cardiovascular risk. *Heart* **102**, 63-68 (2016).
25 De Bacquer, D. *et al.* Prediction of recurrent event in patients with coronary heart disease: the EUROASPIRE risk model: results from a prospective study in 27 countries in the WHO European region-the EURObservational research programme (EORP) of the European society of cardiology (ESC). *European Journal of Preventive Cardiology* **29**, 328-339 (2022).
26 Ishigami, J. *et al.* Acceleration of kidney function decline after incident hospitalization with cardiovascular disease: the Stockholm CREAtinine Measurements (SCREAM) project. *European Journal of Heart Failure* **22**, 1790-1799 (2020).
27 EUROASPIRE. *EUROASPIRE Risk Calculator: Risk of a cardiovascular event in CHD patients*, <https://app.calconic.com/public/calculator/5f6223fab75b14001e1f3c67?layouts=true> (
28 Registry, B. C. Cancer Fact Sheets 2022: Lung cancer. (2024). <https://kankerregister.org/sites/default/files/2024/20240920_BE_CFS_LONG_V1.pdf>.
29 Afifi, A. M. *et al.* Causes of death after breast cancer diagnosis: A US population-based analysis. *Cancer* **126**, 1559-1567 (2020).
30 Van Wilder, L. *et al.* Belgian population norms for the EQ-5D-5L, 2018. *Quality of Life Research* **31**, 527-537 (2022).
31 Doege, D. *et al.* Health-related quality of life in long-term disease-free breast cancer survivors versus female population controls in Germany. *Breast cancer research and treatment* **175**, 499-510 (2019).
32 Koch, L. *et al.* Quality of life in long-term breast cancer survivors–a 10-year longitudinal population-based study. *Acta oncologica* **52**, 1119-1128 (2013).
33 Van Wilder, L. *et al.* A comprehensive catalogue of EQ-5D scores in chronic disease: results of a systematic review. *Quality of Life Research* **28**, 3153-3161 (2019).

© 2026 Busschaert S-L et al. *JAMA Network Open*.
