# Supplementary material for: Cost-Effectiveness of Proton Therapy Compared With Photon Therapy in Breast Cancer
Source: JAMA Netw Open. 2026 Jan 22;9(1):e2554888. doi: 10.1001/jamanetworkopen.2025.54888 (PMC12828626; doi:10.1001/jamanetworkopen.2025.54888)
Supplement: Supplement 2. — Data Sharing Statement [file jamanetwopen-e2554888-s002.pdf]

Data Sharing Statement

Busschaert. Proton Therapy Compared With Photon Therapy—A Cost-Effectiveness Analysis in Breast Cancer. *JAMA Netw Open*. Published January 22, 2026.
doi:10.1001/jamanetworkopen.2025.54888

Data
Data available: No

Additional Information
Explanation for why data not available: All model parameters and assumptions are detailed in the Appendix. Additional information is available upon request.
